# SELSTOC: Self‑Limiting Sternal Tumour of Childhood

**DOI:** 10.5334/jbsr.3820

**Published:** 2025-03-04

**Authors:** Anne Dessent, Bjorn Valgaeren, Yves Lenaerts

**Affiliations:** 1 Department of Radiology, University Hospitals (UZ) Leuven, Gasthuisberg Campus / KU Leuven, Leuven, Belgium

**Keywords:** SELSTOC, pediatric radiology, thoracic wall, ultrasound

## Abstract

*Teaching point:* Creating awareness of self‑limiting sternal tumour of childhood (SELSTOC) in infants with rapidly growing sternal swelling with normal blood results and without trauma and systemic inflammatory diseases.

## Case

**Figure 1:**
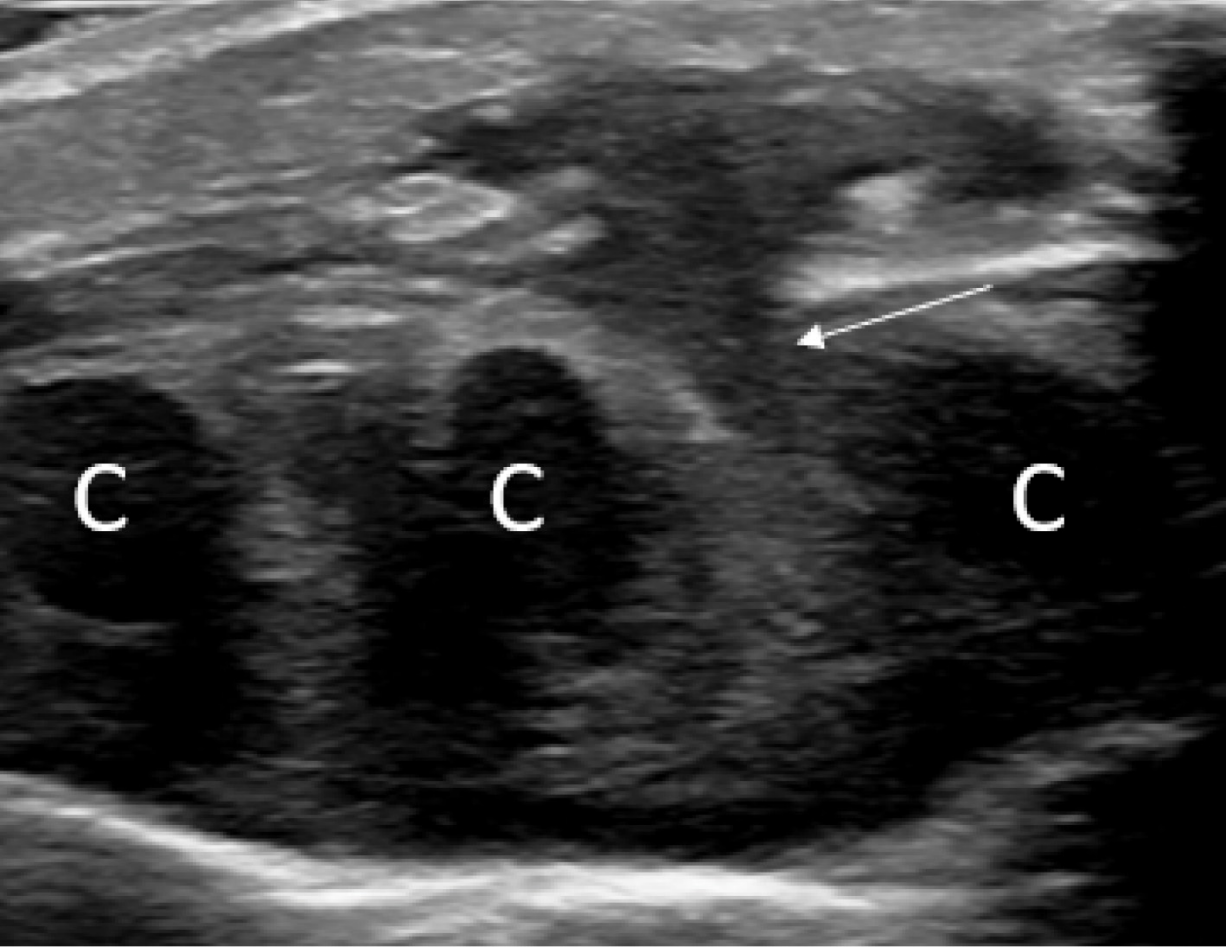
Ultrasound confirmed a soft tissue lesion arising from the space between the fifth and sixth intercostal cartilage, referred to as the ‘dumbbell sign’.

**Figure 2:**
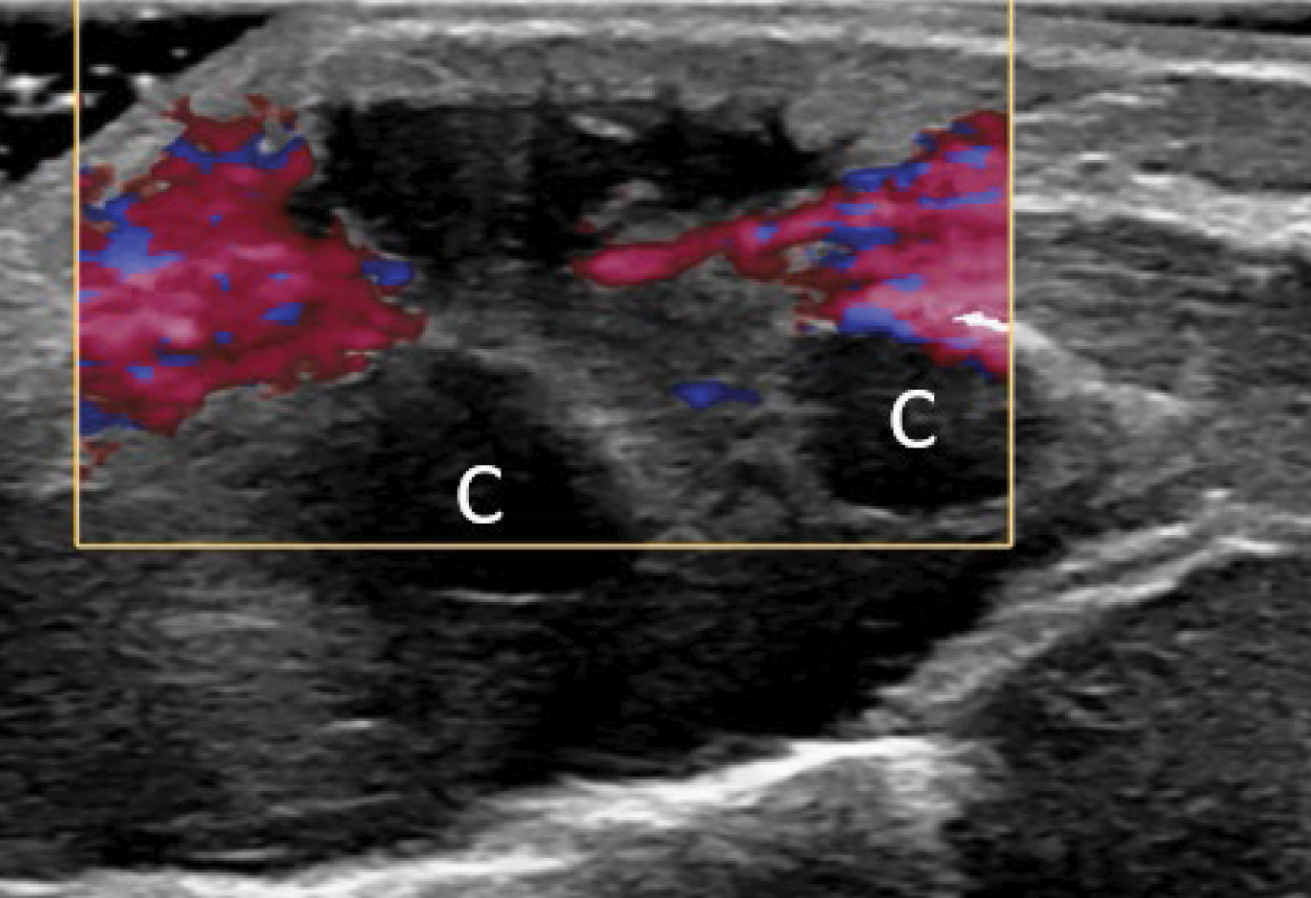
The lesion was surrounded by hyperaemia and hyperechogenicitiy.

An 18‑month‑old girl presented with a painful right‑sided parasternal swelling, which had developed without any known trauma. Blood tests for infection or inflammation were normal. An X‑ray examination was performed and showed no significant abnormalities. The next day, the pain worsened, and she started having trouble breathing. Given the unclear diagnosis, the patient was referred to a tertiary center approximately 7 days after the initial presentation. An ultrasound study confirmed a subcutaneous, well‑delineated, hypoechoic soft tissue lesion 18 mm in diameter. The lesion had a neck (arrow) with a deeper component arising from the space between the fifth and sixth costosternal cartilage (C), referred to as the ‘dumbbell sign’ (Figure 1). Hyperaemia and hyperechogenicity surrounded the lesion (Figure 2).

**Figure 3:**
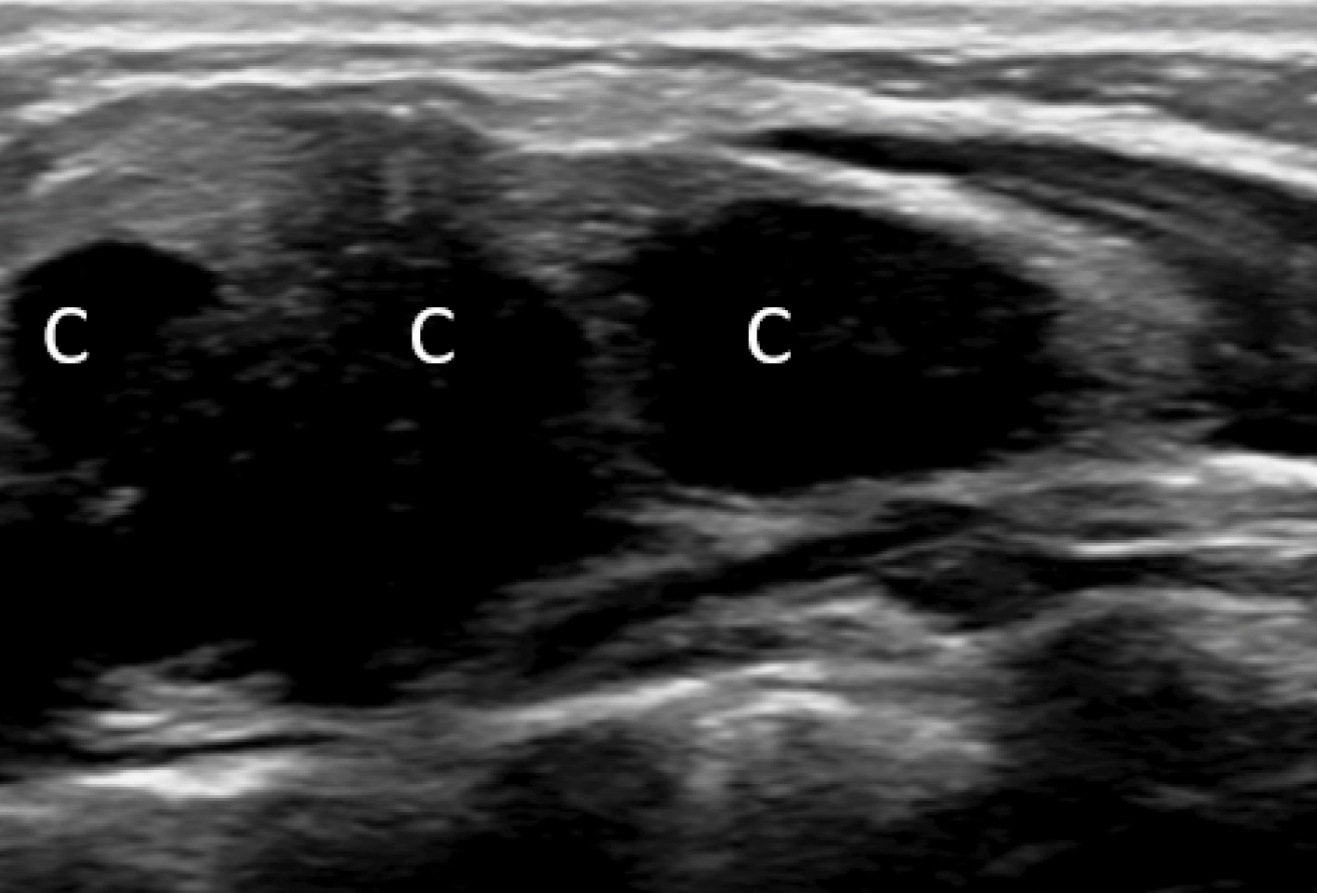
An additional follow‑up three months after the initial presentation showed regression of the pain and swelling.

The diagnosis of self‑limiting sternal tumour of childhood (SELSTOC) was suggested, with a differential diagnosis of osteomyelitis. An additional follow‑up 3 months after the initial presentation showed regression of the pain and swelling (Figure 3), confirming the SELSTOC diagnosis.

## Comment

SELSTOC is a benign, self‑limiting, rapidly growing swelling that occurs in young children. The median age at presentation is 16 months. The aetiology is still uncertain but is thought to be an aseptic inflammatory reaction without a prior infection, trauma or underlying malignancy [1]. Young children are often referred to the emergency department with a recent onset and sometimes painful swelling located presternally and without fever or other clinical signs.

In these children, chest radiography has no additional value because the diagnosis is made by ultrasound. A high‑frequency linear transducer will provide the typical image of a dumbbell‑shaped hypoechogenic mass in the subcutaneous tissue, with expansion at the level of the sternal synchondrosis. There is no associated involvement of the bone or the underlying cartilage [1].

The differential diagnosis includes other benign and malignant lesions, such as recurring chronic multifocal osteomyelitis, haemangioma, lymphatic malformations, Ewing sarcoma, osteosarcoma and metastatic tumours. The combination of young age, rapid growth, typical ultrasound findings and lack of illness should suggest SELSTOC, for which a wait‑and‑see approach is appropriate. If the presentation is unclear or rather atypical, the ultrasound should be supplemented with computed tomography and/or magnetic resonance imaging to rule out the differentials.

## References

[r1] Alonso Sánchez J, Gallego Herrero C, García Prieto J, et al. Self‑limiting sternal tumors of childhood (SELSTOC): a diagnostic challenge. Radiologia (Engl Ed). 2021;63(5):400–405. 10.1016/j.rxeng.2020.04.008. 34625195

